# Exploring Health Care Disparities in Genetic Testing and Research for Hereditary Cardiomyopathy: Current State and Future Perspectives

**DOI:** 10.1055/s-0044-1779469

**Published:** 2024-02-01

**Authors:** Helen Huang, Jay Verma, Valerie Mok, Hareesha R. Bharadwaj, Maen M. Alrawashdeh, Adarsh Aratikatla, Sourav Sudan, Suprateeka Talukder, Minatoullah Habaka, Gary Tse, Mainak Bardhan

**Affiliations:** 1 Department of Medicine, School of Medicine, Royal College of Surgeons in Ireland, University of Medicine and Health Science, Dublin, Ireland; 2 Department of Medicine, Maulana Azad Medical College, University of Delhi, Delhi, India; 3 Department of Medicine Faculty of Medicine, University of British Columbia, Vancouver, British Columbia, Canada; 4 Division of Medical Education, Faculty of Biology Medicine and Health, The University of Manchester, Manchester, United Kingdom; 5 Department of Medicine, Government Medical College, Jammu, Jammu and Kashmir, India; 6 Department of Medicine, Norfolk and Norwich University Hospital, Colney Lane, Norwich, United Kingdom; 7 Tianjin Key Laboratory of Ionic-Molecular Function of Cardiovascular Disease, Department of Cardiology, Tianjin Institute of Cardiology, Second Hospital of Tianjin Medical University, Tianjin, People's Republic of China; 8 Department of Medicine, Kent and Medway Medical School, Canterbury, Kent, United Kingdom; 9 Department of Medicine, School of Nursing and Health Studies, Hong Kong Metropolitan University, Hong Kong, People's Republic of China; 10 Department of Medical Oncology, Miami Cancer Institute, Baptist Health South Florida, Miami, Florida, United States

**Keywords:** hereditary cardiomyopathy, disparities, genetic testing, diversity, ethnic representation

## Abstract

**Background** Hereditary cardiomyopathies are commonly occurring myocardial conditions affecting heart structure and function with a genetic or familial association, but the etiology is often unknown. Cardiomyopathies are linked to significant mortality, requiring robust risk stratification with genetic testing and early diagnosis.

**Hypothesis** We hypothesized that health care disparities exist in genetic testing for hereditary cardiomyopathies within clinical practice and research studies.

**Methods** In a narrative fashion, we conducted a literature search with online databases such as PubMed/MEDLINE, Google Scholar, EMBASE, and Science Direct on papers related to hereditary cardiomyopathies. A comprehensive analysis of findings from articles in English on disparities in diagnostics and treatment was grouped into four categories.

**Results** Racial and ethnic disparities in research study enrollment and health care delivery favor White populations and higher socioeconomic status, resulting in differences in the development and implementation of effective genetic screening. Such disparities have shown to be detrimental, as minorities often suffer from disease progression to heart failure and sudden cardiac death. Barriers related to clinical genetic testing included insurance-related issues and health illiteracy. The underrepresentation of minority populations extends to research methodologies, as testing in ethnic minorities resulted in a significantly lower detection rate and diagnostic yield, as well as a higher likelihood of misclassification of variants.

**Conclusions** Prioritizing minority-based participatory research programs and screening protocols can address systemic disparities. Diversifying research studies can improve risk stratification strategies and impact clinical practice.

## Graphical abstract

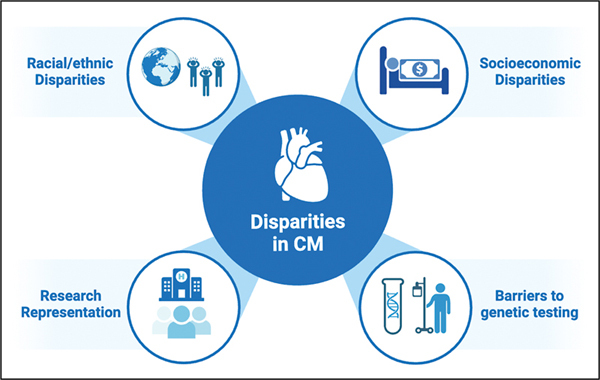

## Introduction

Heredity cardiomyopathies are commonly occurring myocardial conditions affecting heart structure and function, which usually have a genetic or familial association, but in many cases, the etiology is unknown. Cardiomyopathies are linked with significant morbidity and mortality. Generally, inherited cardiomyopathies are managed based on symptom severity, risk of sustained ventricular arrhythmia, and degree of myocardial dysfunction. 1 2 Most treatment strategies are based on the assumption that all patients have the same phenotype, often defined by wall thickness or left ventricular (LV) ejection fraction.

Racial and ethnic disparities in research study enrollment and health care delivery favor White populations and racial/ethnic individuals from higher socioeconomic status, resulting in differences in the development and implementation of the evidence base required for the development of risk stratification tools such as genetic screening. Such disparities have shown to be detrimental. Black patients with hypertrophic cardiomyopathy (HCM) are more likely to suffer from symptomatic heart failure compared with their White counterparts. 3 Studies have shown that cardiomyopathy testing in underrepresented minority populations has a significantly lower detection rate, increased rate of inconclusive test results, and misclassification. 4 5

The advent of precision medicine, however, might address this gap by considering individual patients' genetics, comorbidities, and environmental and lifestyle heterogeneity to select the best strategy for disease prevention and tailored treatment. 6 Providing targeted information can improve the health of individuals and populations and potentially overcome disparities. New tools, such as those that incorporate sociopolitical determinants of health, will be required to describe the cardiovascular health status of individuals and populations. As a result, there is great promise in improving risk stratification, which can be a potential application of precision medicine in cardiology. However, the disproportionate impact of racial and ethnic disparities on the diagnosis of cardiomyopathy remains elusive, and the lack of research could potentially impair developments in inclusive risk stratification of this condition. In this narrative review, we aim to discuss the epidemiology, pathophysiology, genotype, and current diagnostic guidelines of cardiomyopathies and bring forward potential disparities causing limitations in its diagnoses. By raising awareness and putting forward recommendations to improve this gap in the literature, we discuss the practical applications of newer advancements in clinical cardiology.

## Classification, Epidemiology, and Pathophysiology of Cardiomyopathy

**Fig. 1:**
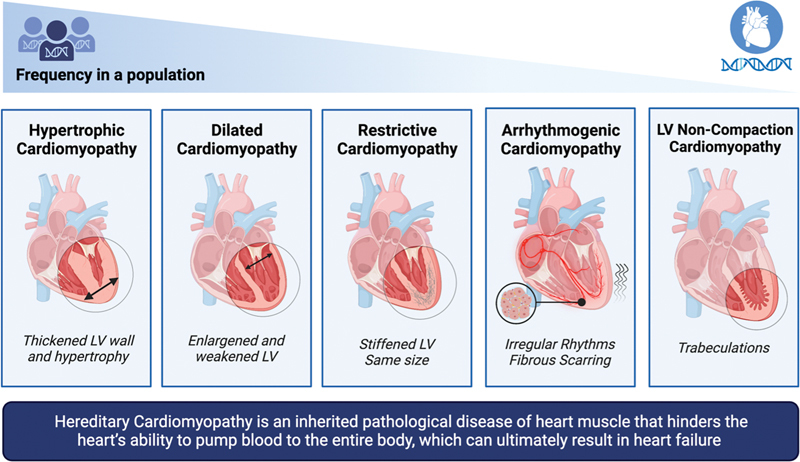
Different types of inherited cardiomyopathies and differentiating characteristics. LV, left ventricular.

Cardiomyopathy is a pathological disease of the heart muscle that hinders the heart's ability to pump blood to the entire body, which can ultimately result in heart failure. 7 The five main types of cardiomyopathy are HCM, dilated cardiomyopathy (DCM), restrictive cardiomyopathy (RCM), arrhythmogenic cardiomyopathy (ACM), and left ventricular noncompaction cardiomyopathy (LVNC) 7 8 ( Fig. 1 ).

The epidemiology of HCM is nonspecifically distributed among the general population and does not target a certain geography, gender, or ethnic group; the prevalence of this disorder is approximately 0.16 to 0.29% in adults. 9 10 The pathologies associated with HCM include left ventricular hypertrophy (LVH) with varying presence of right ventricular hypertrophy, myocardial fibrosis or ischemia, mitral valve insufficiency, flawed cardiac cycle, and obstruction of the left ventricular outflow obstruction (LVOT). 11 Mutations in the *MYBPC3* and *MYH7* genes are seen in about half, whereas *TNNI3* , *TNNT2* , and other genes account for less than 20% of all HCM cases. 12 13

Around 33 to 50% of idiopathic or familial DCM cases are hereditary, which is caused by mutations in over 30 genes that control cardiac muscle function. 14 15 16 The prevalence familial DCM is between 0.04 and 0.4 in the general population, and the incidence is 5 to 7 reported cases per 100,000 people per year. 15 17 Symptoms of familial DCM include fatigue, arrhythmias, dyspnea, and swelling of the lower extremities. The etiology of familial DCM, in addition to genetic inheritance, includes a history of infection, excessive drug and alcohol intake, and coronary artery disease. 14 A commonly implicated gene seen in familial DCM cases is *TTN* , which codes for a protein called titin that plays an essential role in the structure, construction, and signaling of sarcomeres. 18

While the prevalence of familial restrictive cardiomyopathy (FRCM) is not known, it is said to account for 30% of all RCMs, which make up 5% of all cardiomyopathic cases, indicating the rarity of the inherited form. 19 20 The age of onset for familial RCM is highly variable. Patients may have arrhythmias, experience palpitations, or suffer from dizziness, but there are not any specific pathological conditions that lead to the familial form of RCM. 21 The most common genetic defect seen in FRCM is in the gene *TNNI3* , which encodes for cardiac troponin I; however, *MYH7* , *TNNT2* , *TPM1* , *MYL2/3* , and *ACTC1* have also been known to lead to this condition. 22

The frequency of ACM in the general population is between 1:1000 and 1:5000. 23 ACM was previously thought to be a disease localized to northeastern Italy. Still, studies over the last few decades dispel this information, as different haplotypes occur worldwide. 24 ACM has been seen to cause sudden cardiac death in 10 to 15% of the population. 25 Mutations affecting desmosome structure and formation are the most commonly implicated genes contributing to this disorder; these include *PKP2* , *DSP* , *JUP* , *DSG2* , and *DSC2* . 25

Similar to ACM, the data on the prevalence of LVNC is not well known. Various studies have stated that 1 to 15% of the general population suffers from this condition; the disparity in these statistics is due to the imaging modality used to diagnose the LVNC. 26 The etiology of LVNC is diverse: patients have been seen to inherit this in both an autosomal dominant and recessive pattern, as long as an X-linked recessive pattern. 27 The genes affected in this condition are similar to those seen in HCM, as the two most common mutations are in the *MYBPC3* and *MYH7* genes. 27 28

## Genetic Variants of Hereditary Cardiomyopathy

During the last several decades, many genetic abnormalities associated with cardiomyopathy have been uncovered ( Tables 1 and 2 ). HCM is inherited in an autosomal dominant fashion since more than half of the patients have a family history of the condition. A third of DCM patients have family members who also have the condition, which is primarily consistent with autosomal dominant inheritance. However, certain familial instances can also be explained by autosomal recessive or X-linked recessive features. RCM, ARC, and LVNC all have familial occurrences, as well. Genetic studies have allowed researchers to interpret illness-linked mutations in genes situated within the loci. A key finding is that illness genes for different clinical categories overlap.

**Table 1:** Dilated cardiomyopathy- and hypertrophic cardiomyopathy-associated genes

Thick and thin filaments	Cytoskeletal elements	Transmembrane proteins	Nuclear and cytoskeletal proteins	Desmosomes	Miscellaneous
β-Myosin heavy chain *MYH7* α-Myosin heavy chain *MYH6* α-Tropomyosin *TPM1* Cardiac troponin T *TNNT2* Cardiac troponin I *TNNI3* Troponin C, slow *TNNC1* Cardiac actin *ACTC* Myosin-binding protein C *MYBPC3* Titin *TTN*	Filamin C *FLNC* Cypher/ZASP/Lim domain binding 3 * LBD3 a* Vinculin/metavinculin *VCL* Muscle LIM protein *CSRP3* Telethonin – TCAP *TCAP* Nexilin *NEXN* Nebulette *NEBL* α-actinin-2 * ACTN2 a* Laminin α-4 *LAMA4*	Dystrophin * DMD a* β-Sarcoglycan *SGCB* α-Sarcoglycan *SGCA* δ-Sarcoglycan * SGCD a* γ-Sarcoglycan *SGCG* αB-Crystallin *CRYAB* Fukutin * FKTN a*	Desmin * DES a* Lamin A/C * LMNA a* Emerin * EMD a* Tafazzin G4.5 * TAZ a* EYA4 *EYA4* SUR2 * ABCC9 a* Myopalladin * MYPN a*	Plakophilin *PKP2* Desmoplakin * DSP1 a* Desmoglein 2 *DSG2* Plakoglobin * JUP a*	Presenilin-1 * PSEN1 a* Presenilin-2 * PSEN2 a* Four and half LIM protein-2 * FHL2 a* Integrin-linked kinase * ILK a* Acetylcholine receptor * CHRM2 a*

a Genes associated only with dilated cardiomyopathy.

**Table 2:** List of genes associated with restrictive cardiomyopathy, ARC, and left ventricular noncompaction cardiomyopathy

RCM-associated genes		ARC-associated genes		LVNC-associated genes	
β-Myosin heavy chain	*MYH7*	Plakophilin-2	*PKP2*	Tafazzin	*TAZ*
Cardiac troponin T	*TNNT2*	Plakoglobin	*JUP*	Dystrobrevin alpha	*DTNA*
Cardiac troponin I	*TNNI3*	Desmoglein-2	*DSG2*	Homeobox 5 homeobox protein	*NKX2-5*
Myosin-binding protein C	*MYBPC3*	Desmocollin-2	*DSC2*	β-Myosin heavy chain	*MYH7*
Titin	*TTN*	Desmplakin	*DSP*	Cypher/ZASP/Lim domain binding 3	*LDB3*
Myopalladin	*MYPN*	Transforming growth factor – beta 3	*TGFB3*	Cardiac α-actin	*ACTC1*
Myosin light chain 2	*MYL2*	Titin	*TTN*	Cardiac troponin T	*TNNT2*
Myosin light chain 3	*MYL3*	Transmembrane protein 43	*TMEM43*	Myosin-binding protein C	*MYBPC3*
Lamin A/C	*LMNA*	Lamin A/C	*LMNA*	α-Tropomyosin	*TPM1*
Filamin C	*FLNC*	Sodium voltage-gated channel α subunit 5	*SCN5A*	Cardiac troponin I	*TNNI3*
Desmin	*DES*	Alpha-T-catenin	*CTNNA3*	Lamin A/C	*LMNA*
Discodin, CUB, LCCL domain-containing protein 2	*DCBLD2*			Sodium voltage-gated channel α subunit 5	*SCN5A*
Bcl2-associated athanogene 3	*BAG3*			Dystrophin	*DMD*
				Ribosomal S6 kinases	*RPS6KA3*
				Histone-lysin N-methyltransferase	*NSD1*
				Peripheral myelin protein 22	*PMP22*

Abbreviations: LVNC, left ventricular noncompaction cardiomyopathy; RCM, restrictive cardiomyopathy.

### Mutations in Dilated Cardiomyopathy

The most common mutation in DCM is genetic alterations in the *TTN* gene. This gene is responsible for encoding a key protein, titin, which interacts with both thick and thin filaments and provides an elastic force that helps preserve cardiac function. 29 Hence, it would make sense that a mutation affecting a key sarcomeric protein would result in contractile dysfunction. However, mutations are not limited to this gene; studies by Kimura and Chen et al summarized that mutations in over 40 genes can cause hereditary DCM ( Table 1 ). 30 31 Given that 40 to 50% of patients with genetic testing reveal mutations, it is likely there are still undiscovered cardiomyopathy genes. Since then, more research employing genome-wide association study (GWAS) approaches has found unique genetic factors linked with DCM, as exemplified by previous studies that explored different phenotypic markers associated with DCM. 32 33 34 These three studies linked numerous novel single-nucleotide polymorphisms (SNPs) on multiple chromosomes to distinct illnesses, including DCM. Tadros et al discovered that certain SNPs were shown to be reciprocally related to DCM and HCM, demonstrating the dramatic changes in phenotype depending on the type of mutation. In one of the largest GWAS studies on DCM patients, two novel SNPs on chromosomes 3 and 21 were discovered to be linked to the DCM phenotype. 35 Liu et al, on the other hand, used RNA-seq to examine the *SOX* family gene expression levels in patients with DCM and HCM leading to heart failure. 36 They discovered an increase in this cohort's *SOX4* and *SOX8* gene levels. This gene family has been linked to several functions, including cardiac morphogenesis, angiogenesis, and fibrosis, demonstrating the complexities of the underlying molecular mechanisms of hereditary cardiomyopathy.

### Mutations in Hypertrophic Cardiomyopathy

Several of the genes mentioned in Table 1 are associated with HCM, in addition to *MYL2* , *MYL3* , *CAV3* , *JPH-2* , *OBSCN* , and *MYOZ2* . 30 31 As previously mentioned, mutations in *MYH7* and *MYBPC3* are the most common cause of HCM. Mutations in sarcomeric genes are thought to cause various defects, which then cause myosin molecules in the super-relaxed state to shift to the superbound state, leading to increased myocyte contractility and ATP utilization, which triggers a molecular cascade leading to the histological, morphological, and clinical features of HCM. 37

Although heterozygous mutations in the five key sarcomeric genes ( *MYBPC3* , *MYH7* , *TNNT2* , *TNNI3* , and *MYL2* ) are thought to cause more than half of known cases, acknowledging the role of nonsarcomeric genes can have important implications for diagnosis and treatment. 38 39 In Gyftopoulos et al, a GWAS was conducted to further elucidate the role of nonsarcomeric genes and discovered an association between HCM and novel variants in *KMT2C* and *PARD3B* . 40 However, as pertained to most GWAS studies, the underdiagnosis in International Classification of Diseases (ICD)-10 diagnoses confounds the representativeness of samples and should be considered in interpreting novel variants. Aung et al conducted another GWAS to investigate the genetic basis of LV maximum wall thickness, HCM, and the genetic overlap between the two. 34 Of note, *PROX1* , *PXN* , and *PTK2* were discovered as novel risk loci, among others.

### Mutations in Restrictive Cardiomyopathy, ARC, and Left Ventricular Noncompaction Cardiomyopathy

Only a small number of genes have been linked to familial RCM, even though 30% of RCM sufferers have a family history of the condition, which is regarded as a primary disease of genetic origin ( Table 2 ). 41 Examples of some of the genes affected include DCBLD2, which impairs vascular endothelial growth factor signaling leading to abnormal vascular development, which leads to desmin aggregates and disrupts intercalated discs. There are also other genes related to RCM whose pathophysiology remains unknown in this condition, examples include *LMNA* and *MYH7* . 41 The thrust on uncovering novel genetic risk loci has not been nearly as much for RCM as for DCM and HCM. As a result, there have only been a handful of recent studies comprising low-level evidence about its genetic basis, which makes associating the concerned genetic loci with RCM quite fallible. 42 43 Few genes, like RCM, have been strongly linked to ARC ( Table 2 ). 44 Around 50% of ACM patients are estimated to have mutations in the desmosomal genes such as *PKP2* , *JUP* , *DSP* , and *DSC2* . 45 Desmosomes, which are found in the intercalated discs, are responsible for the mechanical coupling of adjacent cardiomyocytes. They also interact with ion channels, gap junctions, and adherens junctions. 45 While there have been more recent studies on the genetic underpinnings of ARC, they are still case reports and lack large-scale GWAS to identify novel risk loci. As for LVNC, the literature is extremely scant, so much so that less than a hundred studies discussing its genetic basis have been published in the last two decades. Nevertheless, certain genetic loci have been robustly associated with LVNC, primarily consisting of sarcomeric genes and certain genes of the Notch signaling pathway, in addition to certain chromosomal abnormalities and mitochondrial gene mutations. 28

## The Role of Genetic Testing in Risk Stratification Strategies

**Fig. 2:**
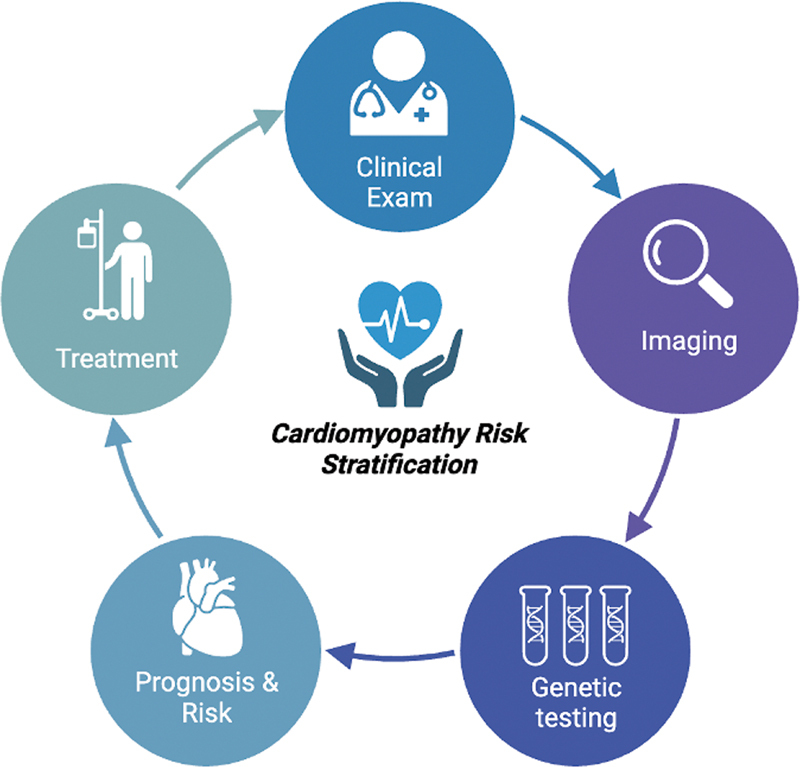
The multidisciplinary approach to diagnose, stratify, and treat cardiomyopathy.

Although there are various modalities to stratify the risk and predict the outcome and prognosis, a multidisciplinary assessment can be carried out with a combination of clinical features, imaging techniques, and genetic testing ( Fig. 2 ). The best way to distinguish between different types of cardiomyopathies is often done using cardiac imaging. The most common modality utilized is transthoracic echocardiography (TTE) because of its widespread availability and cost-effectiveness. 46 LV wall thickness of over 15 mm on TTE is often considered pathognomonic for HCM, as well as features of diastolic dysfunction and LVOT from the anterior motion of the mitral valve. A TTE could also aid in diagnosing DCM by identifying <40% LV ejection fraction and >112% LV end-diastolic volume. 47 However, different imaging modalities may aid in diagnosing different types of cardiomyopathies. For example, an electrocardiogram (ECG) is often the first imaging modality to detect abnormalities such as T-wave inversions and ST-segment elevations in RCM. 48 This modality may also aid in diagnosing ACM, which is often characterized by T-wave inversions and epsilon waves, as well as ventricular ectopy of left bundle branch block morphology. 49 Cardiac magnetic resonance imaging (MRI) with late gadolinium enhancement may yield better anatomical visualization of cardiomyopathy and can add prognostic value to clinical cases by stratifying mortality risk. 50 In RCM, TTE findings of diastolic dysfunction with atrial enlargement could be observed, but cardiac magnetic resonance could be of greater diagnostic value with high specificity to differentiate from other mimics, such as constrictive pericarditis. 51 52

As it currently stands, certain risk factors have been discussed in the literature to help stratify patients. In HCM, for example, a prior aborted cardiac arrest, nonsustained ventricular tachycardia, unexplained syncope, LV thickness >30 mm, abnormal exercise blood pressure, and family history of premature sudden cardiac death are all major risk factors for sudden cardiac death. 9 Moving onto DCM, LVEF, and myocardial fibrosis detected with late gadolinium enhancement have been proposed as methods for risk stratification. 53 However, this still has its pitfalls; for example, the Danish study failed to find an improvement in all-cause mortality using a prophylactic implantable cardioverter–defibrillator in patients with nonischemic systolic heart failure < 35% ejection fraction. 54 This is where genetic testing might help further stratify patients who would benefit from interventions.

Genetic testing is increasingly becoming an important factor in the diagnosis of cardiomyopathy and in delineating rarer types such as ACM and RCM. In addition, genetic testing has also been used recently to predict patient outcomes and stratify the population. Because patients with cardiomyopathies are at a higher risk of suffering from arrhythmogenic outcomes and sudden cardiac death, genetic testing provides diagnostic etiological information that could stratify these outcomes in carriers among family members and affected probands. For example, DCM genes associated with unfavorable outcomes and need intervention include the *LMNA* , *RMB20* , *TTN* , *FLN* , and *PLN* . Patients with these variants are often associated with debilitating outcomes, requiring implantable cardiac defibrillators to prevent sudden cardiac death. 52 Yet, almost 55% of pathogenic variants in DCM are idiopathic and may overlap with variants causing ACM, highlighting the need to implement better classification in a genotypic setting. 55

Similarly, with HCM, the common genes involved are *MYH7* and *MYBPC3* , which have a high degree of major adverse cardiovascular effects. 51 Risk stratification strategies for HCM are most commonly cited as clinical factors such as severity of LVH and family history of HCM could improve yield from genetic testing to up to 60%. The current role of genetic testing is to enable cascade screening approaches with at-risk families, as increasing evidence suggests that screening for a panel of HCM genes could help identify causative variants. However, the rise in rare variants can occur in a given population, creating challenges in determining pathogenic or benign variants of specific alleles. 56

Because RCM, HCM, and LVNC are less common in a population, less research has been done to determine “positive control” genes that could be included in a panel for genetic testing. For example, RCM genes overlap DCM, but case studies exist on delineated less common mutations that have led to sudden cardiac death at an early age. 57 In addition, *TNNT2* is an implicated sarcomeric gene in LVNC potentially involved with cardiogenesis, leading to rapid disease development. Still, it is unknown how these mutations could affect mortality. 58 As such, further research could be considered to better understand the genetic contribution to risk stratification for cardiomyopathy. 59

## Exploring Disparities in Cardiomyopathy from Clinical Practice to Research

### Race-Based Determinants and Ethnic Discrepancies

The diagnosis and risk stratification of hereditary cardiomyopathies, especially HCM and DCM, are riddled with disparities, particularly those pertaining to race and ethnicity. Multiple drivers for ethnicity-based disparities in cardiomyopathy diagnosis and risk stratification exist; consequently, marginalized communities experience a pervasive disadvantage, and greater attention is warranted to unmeasured barriers to care. Primarily, there exists a distinct subset of unique characteristics distinguishing the symptomatology and clinical trajectory of hereditary cardiomyopathies in patients hailing from minority backgrounds, notably exemplified by Black patients, as compared with their White counterparts. For instance, a seminal study evaluating decades worth of data contained within the Sarcomeric Human Cardiomyopathy Registry in the United States reveals that Black patients with hereditary cardiomyopathies were younger at the time of diagnosis, had higher prevalence of NYHA class III or IV heart failure at presentation, had lower rates of genetic testing, and were less likely to have sarcomeric mutations identified by genetic testing. 3 Furthermore, Black patients exhibited a higher propensity for apical-mid and mid-ventricular LVH along with diffuse hypertrophy. Similar diagnostic distinctions in Black populations were also noted in the United Kingdom, where a seminal study exposed that Black patients often manifested abnormal ECG findings and displayed rarer patterns of hypertrophy (concentric and apical hypertrophy being more prevalent in Black populations compared with White populations). Additionally, Black patients presented more frequently with hypertension compared with White counterparts. 60 Hence, the absence of overt presentation with the “classical” features of cardiomyopathies results in diagnostic delays and hampers risk stratification, particularly when health care practitioners are often not trained to recognize such subtleties in minority populations. 61

Compounding this issue is the underrepresentation and disregard of racial and ethnic differences in disease trajectory in the formulation of clinical guidelines. For instance, to date, the American and European guidelines on the diagnosis and management of HCM fail to incorporate the subtleties in disease presentation and outcomes within minority populations. 61 Such disparities result from significant research gaps that persist. Despite the wealth of literature pertaining to disease expression, trajectory, and prognosis of hereditary cardiomyopathies, a notable dearth of data persists concerning these variables in the context of various racial and ethnic cohorts. Research scrutinizing HCM manifestation, disease trajectory, and outcomes across diverse races and ethnic groups, particularly among traditionally underserved populations, including Hispanic/Latin and Black groups, continues to be extremely scarce. 61 62 Furthermore, there exists an absence of comprehensive cardiomyopathy epidemiological registries for minority populations, such as native Americans, Asians, and Jewish Americans. 62 Such prevalent research gaps further worsen ethnic and racial disparities in cardiomyopathy care.

Social disparities further exacerbate these discrepancies. Within the United States, for instance, there is a pronounced disparity in the morbidity and mortality from idiopathic DCM between African Americans and their White counterparts; African Americans are twice as likely to develop the condition and are five times more likely to die from it as compared with White patients. 63 64 These discrepancies likely originate from a greater presence of risk factors (hypertension, diabetes, bronchial asthma), neighborhood and social–environmental risk factors, lack of access to primary care centres, lower levels of educational attainment, and disparities in household incomes within the African American populace. 65 66 Consequently, the culmination of these factors prevents minority populations from accessing specialist care, leading to lower levels of diagnosis of cardiomyopathies. It is unknown how this could affect mortality, and further work may be needed to determine how overcoming racial barriers could assist clinicians in management-related decisions for underrepresented individuals.

### Community-Wide Socioeconomic Disparities

The existence of racial and ethnic disparities in the realm of cardiomyopathy diagnosis and risk stratification is further exacerbated by the concomitant presence of economic disparities. A plethora of research indicates that socioeconomic status exerts a measurable and substantial impact on cardiovascular health care and outcomes. 67 The prompt and accurate diagnosis and risk stratification of hereditary cardiomyopathies hinge upon the provision of high-quality health care, a responsibility that frequently falls upon cardiology multidisciplinary teams rather than individual physicians. Hence, access to specialized care is crucial in achieving these objectives. Patients with low incomes, belonging to lower socioeconomic groups, struggle to access such expert multidisciplinary teams owing to financial constraints; consequently, this translates to a dramatic difference in diagnostic rates and consequent risk stratification compared with patients from higher socioeconomic backgrounds and those that can afford expert care. 68

Current HCM guidelines suggest patients undergo a number of diagnostic investigations, including TTE, 24-hour ECGs, cardiac MRIs, stress echocardiography, and/or cardiopulmonary exercise tests, not only for the initial diagnosis and risk stratification but also for subsequent follow-up. 68 In RCM, ECGs, echocardiograms, cardiac MRIs, and biopsies may be needed to diagnose the condition. 69 Moreover, LVNC diagnostic tests include echocardiography, cardiac MRI, and multidetector computed tomography. 70 The point is that in countries with private health care systems, such as the United States, the uninsured populace (typically corresponding to those with lower socioeconomic statuses) face difficulties accessing such diagnostic modalities. In addition, they also tend to face greater out-of-pocket diagnostic costs as compared with insured individuals. 71 Furthermore, even in countries with national health systems like France, an array of disparities persists concerning access to cardiac diagnostic care, particularly affecting individuals residing in low-income areas who encounter considerable challenges in obtaining timely and adequate access to such services. 68

Notably, the domains of racial and economic disparities seem to be explicitly interlinked in the analysis of hereditary cardiomyopathies. Socioeconomic inequalities tend to affect the minority populace more disproportionately. For instance, an analysis of hereditary cardiomyopathy patients from hospitals situated in Washington, DC revealed compelling disparities among Black patients, who were correlated with a lower likelihood of possessing private health insurance, greater difficulties in paying for out-of-pocket expenses, and a higher likelihood of having a household income of $15,000 or less, displayed significantly rates of cumulative survival and greater disease severity. 72 Consequently, there is an intricate relationship between racial/ethnic and socioeconomic barriers to cardiomyopathy diagnosis and risk stratification. Furthermore, this issue is compounded by the phenomenon of health illiteracy. Rates of health illiteracy, which tend to be greater among patients from socioeconomically disadvantaged backgrounds, further contribute to this issue, as studies have demonstrated its link to the lack of uptake of specialist care. 73 For instance, a seminal study conducted in Australia revealed patients hailing from lower backgrounds displayed lower rates of health care uptake and greater rates of nonadherence to medications, with anxiety and poor mental health being major associated determinants. 73 Such factors further worsen outcomes.

### Barriers to Genetic Testing

Genetic factors are autonomous and enigmatic risk contributors to disparate cardiomyopathy outcomes among individuals of varying genetic ancestries. A recent expert statement from the American Heart Association underscores the efficacy of genetic testing in the comprehensive assessment and treatment of a broad array of hereditary cardiovascular conditions, including cardiomyopathies. 74 However, a dearth of research is dedicated to exploring this phenomenon, especially in minority populations. For example, in a review by Shaboodien et al the authors looked for all studies discussing the genetics of inherited cardiomyopathies in Africa and only found four studies on ACM, one on RCM, and none on LVNC. 75 Furthermore, racial disparities may not solely originate from pathogenic mutations themselves but extend to the clinical expression of the disease, influenced by both intrinsic (biological) and extrinsic factors, such as disparities in socioeconomic status or discriminatory practices within health care systems, as previously elucidated. 76 77 Unfortunately, many patients do not receive genetic testing and would not receive genetic-specific management. Many factors contribute to the inaccessibility of genetic testing, such as limited knowledge of genetics, cost, and lack of insurance coverage. One study in the Netherlands illustrated that patients with asymptomatic disease had difficulties applying for insurance coverage for genetic testing. Better education for patients on the limitations of insurance coverage is important to avoid problems with genetic testing. 78

One major barrier to the efficacy of genetic testing in cardiomyopathy is diagnostic yield. This is indicated as the percentage of patients positively identified to have a genetic component in a cohort of patients identified to have an arrhythmia. The diagnostic yield increases as more genes are tested. 77 An essential factor in genetic testing is the potential misclassification of benign variants and pathogenic mutations, particularly in Black American populations, owing to the limited representation of diverse populations in genome sequencing efforts. 5 The literature demonstrates a disparity in the benefits of genetic testing between White and non-White patient populations, with the former showing a higher diagnostic yield. 77 A seminal investigation systematically assessing the correlation between race and ethnicity and diagnostic yield revealed noteworthy disparities, wherein White individuals exhibited notably higher rates of positive detection and significantly lower occurrences of inconclusive results as compared with underrepresented minorities. 4 77 A plethora of reasons can explain such disparities. Underrepresented minority groups, including Asians, have historically been insufficiently scrutinized in the context of establishing causal connections between genetic discoveries and the diagnosis of cardiomyopathies. This issue of underrepresentation in cardiac genetic studies is compounded with significant research gaps. For instance, there has been a significant lack of research in analyzing and differentiating missense genetic variants for cardiomyopathies from benign variants (which do not cause disease) in minority populations, such as Asians, Native Americans, and Pacific Islanders. 4 77 This paucity in research has translated to lapses in clinical management, as demonstrated by Manrai et al, where five genetic variants formerly deemed causative for HCM ( *TNNT2* , *OBSCN* , *TNNI3* , *MYBPC3* , *JPH2* ) were, in fact, common among individuals of African descent and likely benign. Owing to this erroneous classification, numerous patients of African ancestry harboring these innocuous variants received inaccurate genetic diagnoses 5 ; this may have numerous consequences, such as false reassurances provided to patients, incorrect lifestyle modification advice, and fallacies in clinical management, such overestimation of the benefits of cardioverter–defibrillator implantation. Such erroneous incidents and misdiagnoses have historically extended across multiple cardiomyopathic domains, including HCM and DCM. 75

### Representation in Research and Allelic Variability

Compounding the lack of representation in research are complexities associated with insurance coverage. Although genetic testing is encouraged for the predictive diagnosis of hereditary cardiomyopathies, they may have negative social consequences, such as complexities in accessing insurance coverage. For instance, a seminal study conducted in the Netherlands revealed that 59% of HCM gene carriers reported difficulties while accessing health insurance, with greater difficulties in patients with disease manifestations and symptomatic HCM carriers. In addition, such patients also reported difficulties accessing medical records and paying higher insurance premiums. 78 Similarly, a study evaluating the relationship between cardiovascular genetic screening and insurance coverage policies in the United States reveals significant disparities among different genetic coverage programs; different insurance programs cover different genes for the same condition, causing a lack of uniformity. 79 This circumstance can present distinct challenges for patients hailing from minority backgrounds, given that the genetic causal variants implicated in cardiomyopathies exhibit race-specific disparities, with variants prevalent in minority populations having been inadequately investigated. Consequently, despite the substantial benefits of genetic testing in enhancing diagnosis and risk stratification of hereditary cardiomyopathies, further research is imperative to assess genotype–phenotype associations and their usefulness in diverse patient populations. 76 77 80 In addition to these prospects, more research could be done on less common cardiomyopathies such as ACM, RCM, and LVNC, as disparities in these patient groups are less known.

## Potential Considerations to Bridge Disparities in Hereditary Cardiomyopathies

Addressing these systemic factors will likely have a greater impact on disparities found in cardiomyopathy presentation, severity, and outcomes. The 2018 Heart Failure Society of America guidelines state that pedigree analysis of proband patients should be examined going back at least three generations, and this may be especially important in non-White populations because previous relatives may have been misdiagnosed or not recognized to have cardiomyopathy. 81 Access to genetic testing remains a large barrier for non-White populations. This has detrimental downstream effects as the lack of diverse genetic data causes genetic testing to be less helpful for identifying pathological mutations in non-White populations and instead may be classified as variants of uncertain significance (VUS) or inconclusive. 4 Increasing genetic testing accessibility for minority groups would make genetic testing more clinically helpful in diagnosing cardiomyopathy in these populations; its benefit would be amplified through cascade testing of relatives and build upon their respective ancestry's databases. 82 Beyond the proband patient, testing provides valuable information for the patient's relatives and genetic counselling. Although genetic testing accessibility is only one aspect of health inequality, recognition of more pathological mutations in people of different genetic ancestry would play a role.

Focusing on minority-based and participatory research could also bridge the gap in our understanding of contributing factors leading to disparities in hereditary cardiomyopathy. The socioecological model is a structure emphasizing the many levels of influence impacting patients' behaviors and clinical outcomes. This model takes into account biological and genetic aspects of a patient's risk for disease and places those at risk into the context of community and societal factors to enable clinicians and researchers to understand the variety of factors influencing the outcome of an individual. 83 This model helps visualize important areas that should be targeted to improve ethnic/race disparities in heredity cardiomyopathy. Encouraging the participation of minority patients in clinical trials, cohorts, and genomic studies should be high priority for clinicians. Research in minority ethnic settings with an emphasis on participation from people of diverse ancestry will also likely increase public awareness of the utility of genetic testing. 84 Since most mutations identified in cardiomyopathies are private, there is a great need for further identification and analysis of VUS found more frequently in non-White European ancestry patients. The opposite holds true: benign mutations in non-White patients may be misclassified as pathogenic because they were not previously identified, possibly due to a lack of diverse genetic testing. 5 By increasing recruitment of and matching patients of different ancestry backgrounds across case and control cohorts, such mistakes are less likely to occur. We recommend expanding large-scale genetic studies such as next-generation sequencing in the research context so a more complete picture of both benign and possibly significant variants can be established. Research done in these communities should place additional emphasis on informed consent and ethical principles of research and discussion with those community members from diverse ancestry populations and relevant stakeholders should be initiated prior to beginning study recruitment. 85

As genetic testing is refined, it will become cheaper and quicker to administer, increasing accessibility. Genetic testing might also allow specific mutations to be targeted; for example, Kyriakapoulou et al were able to restore *PKP2* , the most common mutation in ACM, in pluripotent stem cell-derived cardiomyocytes using adeno-associated virus delivery of PKP2. 45 Hence, it might be important to expand our knowledge of the genetic causes of hereditary cardiomyopathies across different ancestries. Furthermore, while genotypes are not yet being used for clinical decision-making in cardiomyopathy, they may represent a promising avenue for estimating prognosis or risk stratification as our knowledge of the pathogenicity of mutations grows. For instance, HCM has strong genetic causal links, making genetic testing information particularly valuable, but because the data have been collected from predominantly White ancestry populations, it is unclear how representative it may for populations of other ancestries. 86 Incorporating VUS into multigene panels, especially when testing diverse ancestral populations, could help clarify their association with disease. In clinics today, even if a pathogenic mutation has not been identified after testing an index patient, non-White European patients diagnosed with cardiomyopathy may still present with (or eventually have) a strong familial pattern and be classified as having idiopathic cardiomyopathy. Its hereditary nature can be further confounded due to phenotypic variation and variable age of symptom onset. As a result, clinicians should educate patients of the possibility of a familial pattern and endeavor to follow up periodically with the patient and their at-risk relatives throughout the life course. 74 This could improve early recognition and intervention of cardiomyopathy and improve clinical outcomes. We would also suggest a lower threshold to offer genetic testing to patients of non-European ancestry, such as in cases of idiopathic cardiomyopathy or cardiomyopathy without identifiable cause.

Finally, considering the utility of bias training in clinical health care could overcome poorer outcomes associated with disparities in testing based on socioeconomic status and race. 87 Inequities in clinical care provision, social and socioeconomic factors, and provider bias may be contributary to ethnic/racial differences in disease expression and poor clinical outcomes. There must be increased awareness and recognition of hereditary cardiomyopathies in minority groups to promote their access to adequate and appropriate treatment. Funding for health care disparities research should also be improved; a suggestion could be to designate parts of funding dedicated to addressing this area of research. 88

## Prospects and Conclusion

Research in the genetics of hereditary cardiomyopathies is a growing area of research and it remains imperative to understand the role of genetic variants in the propensity of disease. Moreover, it has become clinically relevant to consider genetic testing to diagnose the different types of cardiomyopathies to commence early treatment and prevention through high-output screening. However, there remain racial and socioeconomic disparities that prevent the risk stratification of cardiomyopathies. Given the wide phenotypic variation (variable penetrance and expressivity of mutations) in cardiomyopathies, this information cannot be accurately used to predict prognosis or clinical presentation, even if a pathogenic mutation is identified. These issues further translate into clinical research, as the lack of representation can prevent future studies from disseminating clinically significant outcomes among minorities. As a result, future recommendations might consider the utility of addressing systemic issues regarding access to genetic testing and treatment as well as devising minority-based research that could overcome the lack of understanding of cardiomyopathy in underrepresented ethnic groups.
